# Laser Acupuncture Effects on Chronic Pain, Inflammatory Response, and Biochemical and Oxidative Stress Markers in Osteoarthritic Dogs: A Randomized Controlled Trial

**DOI:** 10.3390/ani15172568

**Published:** 2025-08-31

**Authors:** Claudia Gatta, Giovanna Calzaretta, Nadia Musco, Federica Aragosa, Stefano Cavalli, Sara Damiano, Pietro Lombardi, Annunziata Ferrentino, Daria Lotito, Giovanni Della Valle, Maria Elena Pero

**Affiliations:** 1 Department of Veterinary Medicine and Animal Production, University of Naples Federico II, 80137 Naples, Italy; claudia.gatta@unina.it (C.G.); nadia.musco@unina.it (N.M.); stefano.cavalli@unina.it (S.C.); sara.damiano@unina.it (S.D.); pilombar@unina.it (P.L.); daria.lotito@unina.it (D.L.); giovanni.dellavalle@unina.it (G.D.V.); mepero@unina.it (M.E.P.); 2 Olivella Veterinary Clinic, 80137 Naples, Italy; giovanna.calzaretta@gmail.com; 3 Department of Agriculture, University of Naples Federico II, 80137 Naples, Italy; annunziata.ferrentino@unina.it; 4 Department of Pathology, Anatomy and Cell Biology, Columbia University, New York, NY 10032, USA

**Keywords:** osteoarthritis, laser therapy, photo-biomodulation, acupuncture, LAT, cytokines, antioxidants, muscle enzymes, canine

## Abstract

Osteoarthritis in dogs is commonly managed with non-steroidal anti-inflammatory drugs (NSAIDs), nutraceuticals, and other medications aimed at alleviating pain and inflammation. Although NSAIDs are generally considered the most effective option, their use is contraindicated in certain conditions due to adverse side effects. Consequently, there has been growing interest in alternative or complementary therapies, such as acupuncture, laser therapy, and their combination. Laser acupuncture involves stimulating traditional acupuncture points with low-intensity laser light, producing anti-inflammatory, analgesic, anti-edematous, and bio-stimulatory effects. In this study, 19 dogs with osteoarthritis were assigned to two groups: 10 received laser acupuncture treatment (Group T) using a dual-wavelength system delivering continuous and pulsed, synchronized emissions, while 9 served as controls (Group C). After 30 days, only Group T showed significant improvement (*p* < 0.01), with reduced pain and improved joint mobility. Significant reductions in LDH and CPK were observed in Group T, along with changes in inflammatory markers (IL-6 and IL-10, though not TNF-α) and a significant increase in biological antioxidant potential. These preliminary findings indicate that laser acupuncture, applied according to a standardized protocol and adapted to the site of disease, may represent a viable non-invasive therapeutic option for canine osteoarthritis, particularly in cases where pharmacological treatment is contraindicated or ineffective. Further large-scale, randomized controlled trials are warranted to confirm these results and establish long-term efficacy.

## Simple Summary

In dogs with osteoarthrosis, certain conventional therapeutic treatments are not suitable for individuals with specific health conditions. Consequently, in recent years there has been increasing interest in alternative or multimodal therapeutic approaches, including acupuncture, laser therapy, and, in particular, laser acupuncture. Laser acupuncture involves the stimulation of traditional acupuncture points with low-intensity laser light, resulting in anti-inflammatory, analgesic, antiedematous, and bio-stimulatory effects. The present study aimed to evaluate the efficacy of laser acupuncture therapy in 19 dogs with osteoarthrosis, employing a laser system capable of delivering continuous, pulsed, synchronized, and combined emissions. The findings demonstrated improvements in joint mobility and reductions in pain, accompanied by significant changes in inflammatory markers, muscle enzymes, and antioxidant levels, thereby providing robust evidence of the therapeutic benefits of laser acupuncture. Overall, these results support the conclusion that laser acupuncture represents a viable, non-invasive therapeutic option, particularly for dogs in which pharmacological treatment is contraindicated or ineffective.

## 1. Introduction

Osteoarthritis (OA) is a chronic degenerative disease of the joints caused by the deterioration of articular cartilage, and is considered one of the most common causes of pain and disability [1,2]. In dogs, OA is the most common joint disorder [2,3], observed in about 5% of the canine population [4,5,6], and it is clinically manifested by pain, swelling, thickening and crepitation, due to joint effusion or the development of periarticular fibrosis, osteophytosis, and variable reduction in range of motion (ROM). Clinical signs common in multiple joints include lameness, reluctance to jump and climb stairs, difficulty in getting up, stiffness, and decreased activity. In OA, a certain degree of muscle atrophy can be observed due to the inactivity of the subject [7,8]. The etiology of OA is typically multifactorial, involving joint and cartilage wear abnormalities, hip or elbow dysplasia, patella or shoulder dislocation, osteochondritis dissecans, and contributing factors such as obesity, which increases mechanical stress on the joints [9,10]. OA is most observed in senior dogs, with over 50% of cases occurring in those between 8 and 13 years of age. However, approximately 22% of affected dogs are one year old or younger [11]. Beyond age, other predisposing factors include obesity, physical stress, and breed [9,12,13]. One study found that 45% of dogs with OA are of a large breed (with half of those being giant breeds), while only 28% are medium-breed dogs and 27% small-breed dogs [14].

In canine OA, the most common treatments used to alleviate clinical signs include non-steroidal anti-inflammatory drugs (NSAIDs), nutraceuticals, cannabinoids, glucosamine and chondroitin sulfate supplements, and other pain-killers [15,16,17,18,19,20,21,22]. These therapies help reduce pain and inflammation [23,24]. NSAIDs are considered the most effective [24]; however, their use is contraindicated in patients with renal, gastrointestinal, or hepatic diseases. For this reason, alternative approaches such as acupuncture [25,26], laser therapy, or laser puncture may be useful in the management of OA [27,28,29].

Acupuncture is a practice rooted in traditional Chinese medicine (TMC) that involves specific points on the body surface, known as “acupuncture points”). It is an increasingly used treatment for the therapeutic management of pain symptoms and dysfunction associated with musculoskeletal conditions, such as OA [30,31]. There are two main schools of acupuncture, “Eastern and Western”, which differ significantly in terms of philosophy, therapeutic approach, and clinical application [32,33].

Recently, laser acupuncture therapy (LAT) has been proposed as an alternative to traditional acupuncture, eliminating the need for needle insertion. In LAT, traditional acupuncture points are stimulated using low-intensity laser light, a non-invasive, painless, and safer approach compared to needle-based therapy [34,35].

Laser therapy uses penetrating photon energy, known as photo-biomodulation (PBMT), to induce physiological and biochemical changes within the target tissues, resulting in therapeutic benefits such as pain and inflammation relief, wound healing, and tissue regeneration [36]. The main therapeutic effects of laser light used in physiotherapy and veterinary rehabilitation include anti-inflammatory, analgesic, anti-edematous, and bio-stimulating actions. These effects stem from biological responses induced by laser radiation at the cellular and tissue level and contribute to pain reduction without side effects [37]. Laser acupuncture therapy refers to a “photonic stimulation of acupuncture points and areas to promote therapeutic effects similar to those of classical acupuncture and related therapies, along with the benefits of photo-biomodulation” [38].

The interactions between lasers and tissues, as well as the physical properties of the laser beam, are influenced by skin characteristics, including thickness (which affects the degree of beam attenuation), color (which determines the extent of light absorption), and the presence of hair (which promotes the reflection of the laser beam) [39]. Laser–tissue interaction produces photochemical, photothermal, and photomechanical effects: (1) Photochemical effects, resulting from the absorption of specific wavelengths by endogenous molecules, can trigger complex biological processes such as enzymatic activation, ATP production, membrane stabilization, and increased synthesis of DNA and RNA, proteins, growth factors, cytokines, and cell mitosis [40]. (2) Photothermal effects, caused by threading due to the dissipation of absorbed energy, lead to the activation of the local microcirculation. This promotes improved nutrient delivery and the removal of metabolic waste, contributing to anti-inflammatory, analgesic, and anti-edematous effects [41]. (3) Photomechanical effects, resulting from photothermal mechanism, induce reversible changes in tissue structure and result in both analgesic and bio-stimulator actions [42]. This interaction also enhances lymphatic peristalsis, facilitating the drainage of inflammatory edema and the reactivation of microcirculation, thereby improving tissue oxygenation [27,43].

In this study, a diode or semiconductor laser with a wavelength between 650 and 980 nm was used. The device features a continuous and pulsed emission system, combining two synchronized sources with different wavelengths capable of penetrating deep into the tissues. The synchronization of emissions synergistically enhances the therapeutic effects compared to using each wavelength individually [44]. We applied LAT to Western-approach acupuncture points, which focus on reducing inflammation and chronic pain, aligning with the principles of integrative medicine [33,45,46].

Although some studies exist on laser acupuncture in veterinary medicine [47,48,49], its effectiveness in dogs with OA remains to be elucidated.

The aim of this study was to evaluate the effectiveness of laser acupuncture in reducing pain and improving joint function in dogs with OA by analyzing its metabolic effects, its oxidative stress markers, and r-associated pain symptom reduction.

## 2. Materials and Methods

The present prospective clinical trial was conducted from January 2025 to May 2025. In total, 19 dogs (11 males and 8 females; mean age 9.3 ± 3.3 years old; mean body weight 21.4 ± 11.3 kg) were included in the trial. The animals were from a client-owned referral population of the Veterinary Teaching Hospital, Department of Veterinary Medicine and Animal Productions (University of Naples Federico II). The study protocol was approved by the Ethical Animal Care and Use Committee of the University of Naples Federico II (protocol code PG/2025/0069656).

The study population consisted of client-owned dogs with a confirmed diagnosis of OA based on medical history, clinical examination, and imaging. Only dogs classified as graded 3 (severe), according to Pollmeier et al., 2006 [50], were included in the trial. All procedures were performed for diagnostic and therapeutic purposes in accordance with European Directive 2010/63/EU [51]. In addition, all procedures were carried out after informing the authors and obtaining their consent.

**Figure 1:**
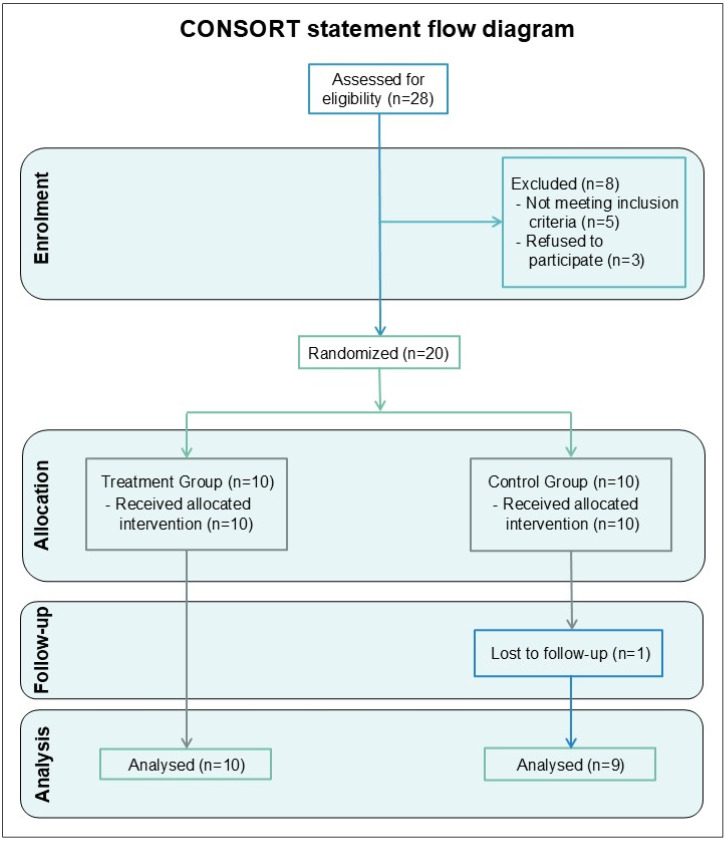
CONSORT statement flow diagram.

No dogs with comorbidities were included in the trial. After enrollment, the dogs were assigned to two groups (control group n = 9 and treated group n = 10) according to a randomized complete block design, using an online randomization tool (https://www.randomizer.org). The patients did not receive any pharmacological treatment to manage clinical signs (such as NSAIDs and nutraceuticals) for at least 2 months prior to and throughout the duration of the trial. Group allocation was concealed from the owners and a double-blind design was employed. A total of 28 dogs were assessed for eligibility, of which 5 were excluded because they did not fall within the inclusion criteria, and the owners of 3 dogs declined participation in laser therapy. One dog was excluded during the study period due to loss of follow up (Figure 1).

The treated group (n = 10) received laser acupuncture therapy, while dogs in the control group (n = 9) received a simulation of a laser acupuncture session. Acupuncture laser therapy uses a dual-wavelength, high-power infrared laser (Multiwave Locked System, MLS^®^ laser, M-VET, ASA laser srl, Arcugnano (VI), Italy). The group receiving laser-perforated Western acupuncture points were treated with a handheld optical instrument according to its differentiation from the practice of traditional Chinese medicine. The control group did not receive any therapy with pharmacological or nutraceutical treatments. Also, they did not receive any LAT treatment, but a simulation using a sham device (non-emitting probe) was performed.

### 2.1. Laser Protocol and Laser Acupuncture Sessions

The treated group received 10 laser acupuncture sessions in 4 weeks. During the first week, three sessions were administered on alternate days, followed by two sessions per week for the remaining three weeks. Each section included both laser treatment and laser acupuncture.

For the laser treatment, areas of spinal edema, spinal inflammation, and trigger points were targeted, including the stifle joints, elbow and iliopsoas muscles, and specific pain points, during both the acute and chronic stages (Table 1 and Table 2). The handheld optical instrument used for the trigger has a beam diameter of 2 cm, a beam divergence of 0.03 rad, and spot area of 0.125 cm^2^.

The Western acupuncture points [46] used in all subjects were SP 6, LI 4, LV 3, ST 36, and GV 20. Each point had certain parameters, such as Noiger 1168 Hz for 15 s, energy 258 J, 20.66 J/cm^2^, and intensity 1% (Table 3). The handheld optical instrument used for acupuncture has a beam diameter of 4mm, a beam divergence of 0.84 rad, and spot area of 3142 cm^2^.

Subsequently, based on the localization of the osteoarthritis, the laser acupuncture points used were pre-set by the machine program, without altering the parameters [46].

### 2.2. Clinical Evaluation

The health status of all dogs was assessed through clinical examination and complete blood count analysis. These evaluations were performed by the same veterinarian, both before starting treatment (day 0) and after 30 days and at the end of the laser acupuncture sessions, without knowledge of the group assignments. At each session, a clinical evaluation was conducted to evaluate lameness, including pain during handling and palpation, joint swelling, and range of motion (ROM). During the initial evaluation [50], the most severely affected leg was identified, and scores for lameness, discomfort on manipulation and palpation, ROM, and joint swelling were assigned to that limb.

For all dogs before the enrollment (day 0) and at the end of laser acupuncture sessions (day 30), the Helsinki Chronic Pain Index (I-HCPI) was used to assess the owners’ perception of pain. Like the original I-HCPI, the Italian version consisted of 11 questions concerning the dog’s mood, vocalization, willingness to walk, trot, gallop, play, and jump, and ease of lying down, getting up, moving after a long rest, and moving after intense activity. Owners answered each question on a 5-point descriptive scale. The single answers were then tied to a value from 0 to 4 and summed to give a total index score from 0 to 4 [52].

### 2.3. Blood Sampling and Analyses

Blood samples were collected from fasting dogs at the enrollment (day 0) and at the end of the last laser treatment (day 30). The samples were placed in plastic tubes and transported to the laboratory (within 1 h). Serum was obtained by centrifugation at 3000× *g* for 15 min, divided into three aliquots, and frozen at −80 °C.

Serum was analyzed for blood chemistry using an automatic biochemical analyzer (AMS Autolab, Diamond Diagnostics, West Point, UT, USA) with reagents from Spinreact (Girona, Spain) to determine lactic dehydrogenase (LDH) and creatine phosphokinases (CPKs).

The effects of LAT on oxidative status were assessed by measuring the serum levels of the reactive oxygen metabolite derivates (d-ROMs), and biological antioxidant potential (BAP) was measured in serum samples. d-ROMS reflect the presence of free alkoxyl and hydroperoxyl radicals derived from hydroperoxides, while BAP measures the chemically active antioxidant capacity of the plasma. Reagents from Diacron International s.r.l. (Grosseto, Italy), validated for use in canine species, were used for the assessments [53].

Inflammatory status was evaluated by measuring the serum level of canine interleukins 6 (IL-6) and 10 (IL-10) and canine tumor necrosis factor alpha (TNF-α), using Elisa kits from Genorise (Philadelphia, PA, USA). The TNF-α had a detection range of 1–2200 pg/mL, with intra- and inter-assay coefficients of variation (CV) of <7 and <9%, respectively. The detection ranges for IL-6 and IL-10 were 50–3200 pg/mL and 25–1600 pg/mL, respectively, with intra- and inter-assay CVs of <6 and <9%. Absorbance was assessed at 450 nm with a microplate reader (GDV DV 990BV4 programmable MPT reader, Agilent Technologies, Santa Clara, CA, USA).

### 2.4. Statistical Analysis

Statistical evaluation was performed by Wilcoxon’s matched pairs signed-rank test using JMP software (JMP^®^, Version 14, SAS Institute Inc., Cary, NC, USA, 1989–2021). Differences were considered statistically significant at *p* < 0.05.

Lameness evaluation data were analyzed by one-way ANOVA (JMP software, SAS Institute, NC, USA) using the model

yijk=Gi+Tj+G×Tij+εijk

where y is the dependent variable, μ is the mean, G is the group effect (i = control, treated), T is the time effect (j = 0, 30), G × T is the first level of interaction, and ε is the error effect.

When significant differences were found in the ANOVA, means were compared using Tukey’s test.

## 3. Results

**Figure 2:**
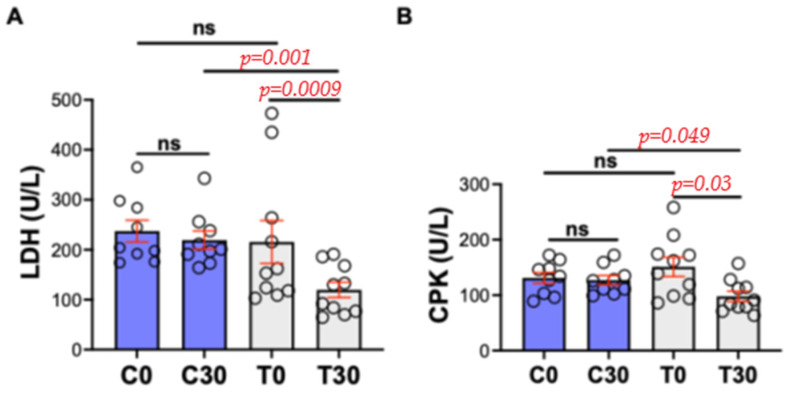
Muscle enzyme in dogs in control and treated groups. C0 = control group at time 0; C30 = control group at time 30; T0 = treated group at time 0; T30 = treated group at time 30. ns = not significant. (**A**) LDH = Lactic dehydrogenase. (**B**) CPK = Creatine phosphokinase.

The statistical approach used in this study was based on comparisons between the control and treated groups at two time points: day 0 and day 30. Concerning the muscle enzyme parameters, the Wilcoxon test showed a statistically significant difference between C30 and T30 for both LDH (*p* = 0.001; 219.3 and 119.6 U/L for C30 and T30, respectively) and CPK (*p* = 0.049; 127.0 and 97.9 U/L for C30 and T30, respectively). Highly significant differences were also detected compared T0 vs. T30 for LDH (*p* = 0.0009; T0 215.7 and T30 119.6 U/L) and CPK (*p* = 0.03; 151.1 and 97.9 U/L for T0 vs. T30, respectively), while no statistical differences were registered for the other comparison (C0 vs. C30 and T0 vs. C0, respectively) (Figure 2).

**Figure 3:**
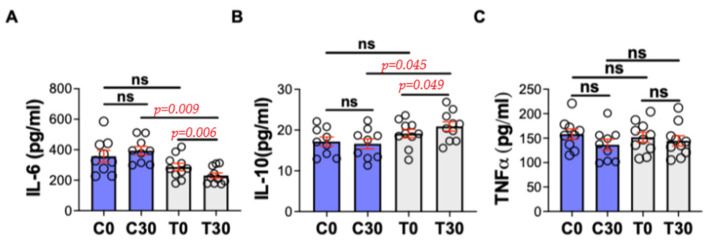
Interleukins and TNF-α in dogs in control and treated groups. C0 = control group at time 0; C30 = control group at time 30; T0 = treated group at time 0; T30 = treated group at time 30. ns = not significant. (**A**) IL-6 = pro-inflammatory cytokine 6; (**B**) IL-10 = anti-inflammatory cytokine 10; (**C**) TNF-α = Tumor Necrosis Factor.

The interleukin analyses showed significant differences at times C30 vs. T30 for IL-6 (*p* = 0.009, 393.3 for C30 and 230.9 pg/mL for T30) and T0 vs. T30 (*p* = 0.006, 286.4 and 230.9 pg/mL). IL-10 was significantly different (*p* = 0.049) compared T0 vs. T30 (19.4 and 20.9 pg/mL) and C30 vs. T30 (16.6 and 20.9 pg/mL, *p* = 0.045). However, comparing times C0 to C30 and T0 to C0, we did not highlight any statistical significance. For TNF-α, no statistical differences were detected (Figure 3).

**Figure 4:**
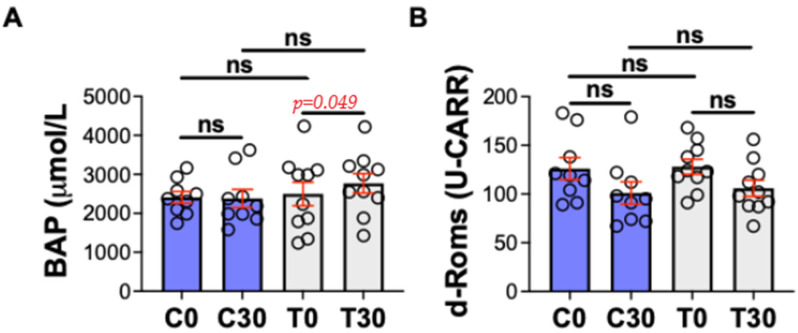
Oxidative stress markers in dogs in control and treated groups. C0 = control group at time 0; C30 = control group at time 30; T0 = treated group at time 0; T30 = treated group at time 30. ns = not significant. (**A**) BAP = biological antioxidant potential; (**B**) d-ROMs = reactive oxygen metabolites derivates.

The oxidative stress markers showed a significant result only for BAP, with *p* = 0.049, comparing T0 vs. T30 (2498 vs. 2770 mmol/L), while no differences were detected for D-Roms (Figure 4).

Despite all dogs falling into the grade 3 (severe) osteoarthrosis classification at the end of the experiment, according to the Helsinki evaluation test (I-HCPI), dogs of group C showed a significant (*p* < 0.01) improvement after 30 days of treatment, whereas no differences were found for the control group (Table 4).

## 4. Discussion

Clinical data showed a significant improvement in subjects treated with LAT compared to the control group, with a clear reduction in lameness pain on palpation, and joint movement restriction. The I-HCPI questionnaire administered to owners revealed a marked decrease in perceived pain and an overall improvement in behavior and mobility in the treated group, while the control group showed no significant changes. These findings are consistent with the literature: LAT has shown analgesic, anti-inflammatory and regenerative properties in various musculoskeletal disorders in both human and veterinary studies [25,26,36]. Its mechanism of action involves mitochondrial stimulation, modulation of the immune response, and improvement in microvascular circulation, with documented effects also on muscle tone and autonomic balance. A meta-analysis on athletes with muscle injuries showed that acupuncture was significantly more effective in reducing pain than standard treatments such as physiotherapy and massage [54]. In addition, a significant improvement in the pain response threshold and a reduction in joint edema was observed in a murine model after treatment with LAT, highlighting the effectiveness of the technique in pain control and cartilaginous protection as well [29].

In parallel to clinical improvement, a significant modulation of some inflammatory biomarkers was observed in the treated group. Specifically, we observed a significant reduction in the cell damage markers LDH and CPK, suggesting decreased secondary muscle tension or inflammation. These findings are consistent with previous studies in patients treated with traditional acupuncture, which reported long-term reductions in muscle damage and inflammation in the context of athletic injuries, including modulation on markers such as CPK and LDH [54]. Furthermore, in treated dogs, we observed a significant decrease in IL-6 (a pro-inflammatory cytokine) and an increase in IL-10 (an anti-inflammatory cytokine). Cytokines play a key role the regulation of inflammation and host defense against bacterial infections, and may also serve as diagnostic markers in various disease conditions [55,56]. The benefits observed in dogs treated with LAT, which were absent in the control group, confirm the positive effects of LAT on immune and inflammatory responses, as previously reported in studies evaluating the use of nutraceuticals [18] and traditional low-level laser therapy (LLLT) treatments [57]. In a study conducted in rats, LLLT was shown to be effective in reducing IL-1β, TNF-α, and MMP-13 [57], although the effects on IL-6 were variable. In our study, the significant increase in IL-10 observed in the treated group is consistent with the findings of Shi and colleagues [58], who also identified a role of another anti-inflammatory cytokine, IL-13, in modulating the chronic inflammatory response. This suggests a potential systemic anti-inflammatory effect of LAT. However, since the *p*-values are close to the threshold for significance, future studies with larger populations will be needed to confirm the true ability of photo-biomodulation to modulate IL-10 levels. Mantineo and colleagues [58] also demonstrated that the use of low-intensity lasers at 830 nm, especially in continuous mode (CW), can modulate the expression of inflammatory cytokines, such as IL-6, IL-1β, and TNF-α in inflamed muscle tissues. Their research showed that photo-biomodulation exerts its effect by improving mitochondrial metabolism through the activation of cytochrome c oxidase, leading to a reduction in the inflammatory response [58]. In traditional acupuncture therapies, modulation of pain and inflammation has also been observed in humans, with several clinical studies and meta-analyses demonstrating a reduction in pro-inflammatory cytokines, such as IL-1β and TNF-α, along with an increase in the production of anti-inflammatory mediators [59,60]. In our study, TNF-α did not show relevant changes between groups. This suggests that the systemic anti-inflammatory effect of LAT, although present, may not involve all major pro-inflammatory cytokines. Previous studies have reported that TNF-α has transient secretion dynamics and very low plasma concentrations in chronic models, which may make it difficult to detect significant changes in clinical studies with a small sample size [55,57,58,59,60]. This finding is also consistent with prior evidence that TNF-α is a pro-inflammatory cytokine primarily involved in initiating acute inflammatory responses rather than sustaining chronic or moderate immune modulation. TNF-α has a very short half-life in circulation (on the order of minutes), which increases the likelihood that transient peaks may have occurred outside our sampling windows and therefore remained undetected in our analysis [61,62]. By contrast, IL-6 and IL-10 are known to have more sustained systemic profiles and play major roles in balancing pro- and anti-inflammatory responses.

Their significant modulation in our study supports the conclusion that the immune response was primarily mediated through these cytokines rather than TNF-α. Furthermore, the lack of statistical significance could indicate a lower responsiveness of TNF-α to photo-biomodulation compared to IL-6 and IL-10 [58]. This suggests that effective modulation of TNF-α may require different treatment protocols in terms of dosage, duration, or frequency to achieve clinically relevant effects.

For these reasons, and given the absence of significant differences in our dataset, we consider that further analyses of TNF-α are unlikely to provide additional meaningful insights. One aspect of particular interest was the behavior of markers associated with oxidative stress. In the treated group in our study, there was a significant increase in BAP, an index of endogenous antioxidant capacity, while the d-ROMs test, which measures the concentration of free radicals, did not show significant changes. This discrepancy may suggest that the treatment effectively activated physiological antioxidant defenses but did not substantially reduce the overall oxidative load in the short term. These findings support the hypothesis that LAT can stimulate endogenous antioxidant mechanisms, although this response may not be sufficient in the short term to fully counteract the systemic oxidative stress in subjects with OA. This interpretation is consistent with findings from animal studies which have shown that acupuncture can increase the expression of antioxidant enzymes such as SOD, catalase, and glutathione peroxidase, while concurrently downregulating pro-oxidant and pro-inflammatory pathways [54]. Indeed, oxidative stress has been identified as a key factor in the progression of cartilage damage and chronic pain [63]. Although the increases in IL-10 and BAP reached statistical significance (*p* = 0.045 and *p* = 0.049, respectively), their proximity to the conventional threshold suggests a risk of type I errors, particularly given our limited sample size. For this reason, these findings should be interpreted with caution and considered preliminary. Nonetheless, the consistent modulation of IL-6 and IL-10 supports the biological plausibility of a systemic anti-inflammatory effect of LAT, which warrants confirmation in future studies with larger cohorts and extended follow-up periods.

In this study we quantified the owner’s perception of their dog’s pain, discomfort, and functional limitation through the HCPI psychometric questionnaire. The scale further confirmed the validity of the treatment, showing a statistically significant improvement during the course of this study, as reported in previous studies [28,64,65]. The pain scales in our study population have also been recommended for use in the assessment of canine OA in a recent systematic review [66] and in the WSAVA guidelines [67].

## 5. Conclusions

The results of this study support the beneficial effects of LAT on the welfare of animals affected by OA, improving joint health and reducing pain. When applied according to a standardized protocol and tailored to the affected sites, LAT demonstrated greater efficacy than the control treatment in managing canine osteoarthritis. Clinical improvements were associated with significant changes in inflammatory, muscular, and antioxidant markers, suggesting a targeted systemic immunomodulatory and anti-inflammatory effect. These preliminary results reinforce the idea that LAT may be a viable non-invasive therapeutic alternative, particularly useful in those subjects where drug therapy is contraindicated or ineffective. The integrated approach to physical, nutraceutical, and pharmacological therapies can open new perspectives in the management of OA and chronic pain in veterinary medicine. Future studies involving larger sample sizes and extended follow-up periods will be essential to confirm LAT’s efficacy and define its optimal role within multimodal therapeutic protocols.

## 6. Limitations of the Study

The present study has several limitations: (1) the small sample size may limit the statistical power for detecting significant differences in some other markers; (2) the follow-up was limited to 30 days, ending with the conclusion of the therapeutic cycle, and did not include long-term evaluations; (3) although the protocol was standardized in terms of energy parameters, it could be further customized based on the location and severity of the lesion; and (4) the lack of objective instrumental evaluations (e.g., force plate, thermography) limits precision in the biomechanical evaluations related to functional improvement.

## Figures and Tables

**Table 1:** Laser parameters concerning skin color characteristics in the acute stage of osteoarthritis.

Trigger Points Acute								
	Clear Skin				Dark Skin			
	*Intensity*	*Time*	*Energy*	*Dose*	*Intensity*	*Time*	*Energy*	*Dose*
Spine, Shoulder, Elbow, Pelvis, Iliopsoas, Stifle	20%	13 s	4.55 J	1.52 J/cm^2^	10%	24 s	4.43 J	1.48 J/cm^2^
**Acute Stage**								
	**Clear Skin**				**Dark Skin**			
	*intensity*	*time*	*energy*	*dose*	*intensity*	*time*	*energy*	*dose*
Spinal Edema	60%	31 s	73.20 J	4.07 J/cm^2^	50%	36 s	73.12 J	4.06 J/cm^2^
Spinal Inflammation	40%	1.59 min	81.04 J	4.50 J/cm^2^	30%	2.37 min	81.02 J	4.50 J/cm^2^

**Table 2:** Laser parameters concerning the skin color characteristics in the chronic stage of osteoarthritis.

Trigger Points Chronic								
	Clear Skin				Dark Skin			
	*Intensity*	*Time*	*Energy*	*Dose*	*Intensity*	*Time*	*Energy*	*Dose*
Spinal, Shoulder, Elbow, Pelvis, Iliopsoas, Stifle	30%	12 s	6.18 J	2.06 J/cm^2^	20%	17 s	5.94 J	1.98 J/cm^2^
**Chronic Stage**								
	**Clear Skin**				**Dark Skin**			
	*intensity*	*time*	*energy*	*dose*	*intensity*	*time*	*energy*	*dose*
Spinal Edema	60%	31 s	73.20 J	4.07 J/cm^2^	50%	36 s	73.13 J	4.06 J/cm^2^
Spinal Inflammation	50%	2.08 min	108.72 J	6.04 Jcm^2^	36%	2.38 min	108.13 J	6.01 J/cm^2^

**Table 3:** Western acupuncture points used for laser acupuncture.

Acute Cervical Paralysis	Acute Lumbar Paralysis	Acute Sacral Paralysis
BL 10, BL 11, GB 20, GV 16, GV 15, GV 14, GV 13, SI 15, TH 15	BL 23, BL 28, BL 39, BL 40, Bai Hui, ST 36, LI 10, GB 29	BL 23, BL 28, BL 31, BL 32, BL 33, BL 34, Bai Hui, ST 36, LI 10

**Table 4:** Helsinki score evaluation of dogs in control and treated groups.

I-HCPI		
C	20.33	
T	24.25	
	C	T
Day 0	19.89B	29.90A
Days 30	20.78B	18.60B
P group	0.04	
P time	0.009	
P group × time	0.003	
RMSE	5.77	

HCPI = Helsinki Chronic Pain Index; C = control group; T = treated group. P group = group effect; T group = time effect; P group × time = interaction between group × time. RMSE = root mean square error. A, B *p* < 0.01.

## Data Availability

The data presented in this study are available on request from the corresponding author.
